# Reactive oxygen species‐induced changes in glucose and lipid metabolism contribute to the accumulation of cholesterol in the liver during aging

**DOI:** 10.1111/acel.12895

**Published:** 2019-01-04

**Authors:** Eunhui Seo, Hwansu Kang, Hojung Choi, Woohyuk Choi, Hee‐Sook Jun

**Affiliations:** ^1^ College of Pharmacy and Gachon Institute of Pharmaceutical Science Gachon University Incheon Republic of Korea; ^2^ Lee Gil Ya Cancer and Diabetes Institute Gachon University Incheon Republic of Korea; ^3^ Division of Life Sciences Korea University Seoul Republic of Korea; ^4^ Gachon Medical Research Institute, Gil Hospital Incheon Republic of Korea

**Keywords:** aging, cholesterol, lipid metabolism, liver, reactive oxygen species

## Abstract

Aging is a major risk factor for many chronic diseases due to increased vulnerability to external stress and susceptibility to disease. Aging is associated with metabolic liver disease such as nonalcoholic fatty liver. In this study, we investigated changes in lipid metabolism during aging in mice and the mechanisms involved. Lipid accumulation was increased in liver tissues of aged mice, particularly cholesterol. Increased uptake of both cholesterol and glucose was observed in hepatocytes of aged mice as compared with younger mice. The mRNA expression of GLUT2, GK, SREBP2, HMGCR, and HMGCS, genes for cholesterol synthesis, was gradually increased in liver tissues during aging. Reactive oxygen species (ROS) increase with aging and are closely related to various aging‐related diseases. When we treated HepG2 cells and primary hepatocytes with the ROS inducer, H_2_O_2_, lipid accumulation increased significantly compared to the case for untreated HepG2 cells. H_2_O_2_ treatment significantly increased glucose uptake and acetyl‐CoA production, which results in glycolysis and lipid synthesis. Treatment with H_2_O_2_ significantly increased the expression of mRNA for genes related to cholesterol synthesis and uptake. These results suggest that ROS play an important role in altering cholesterol metabolism and consequently contribute to the accumulation of cholesterol in the liver during the aging process.

## 1 INTRODUCTION

Aging refers to a state in which homeostasis can no longer be maintained due to structural changes or dysfunction and the organism becomes vulnerable to external stress or damage (Lopez‐Otin, Blasco, Partridge, Serrano, & Kroemer, 2013). Aging is a major risk factor for most chronic diseases such as cerebrovascular disease, neurodegeneration, chronic obstructive sleep apnea, cancer, and diabetes (Bonomini, Rodella, & Rezzani, 2015).

The production of reactive oxygen species (ROS) is progressively increased in aging and is one of the key factors in cellular damage. It is known that ROS, including free radicals and peroxides, adversely affects cells and tissues and causes an imbalance in the biological system (Agarwal, Saleh, & Bedaiwy, 2003; Valko, Morris, & Cronin, 2005), contributing to the development of many aging‐related diseases (Beckman & Ames, 1998; Chandrasekaran, Idelchik, & Melendez, 2017; Sohal, 2002). In addition, oxidative stress plays an important role in hepatic disease (Cichoz‐Lach & Michalak 2014). Aging increases fibrotic responses and is also associated with the development of a variety of liver diseases including nonalcoholic fatty liver disease and alcoholic liver disease (Kim, Kisseleva, & Brenner, 2015). In particular, the prevalence of nonalcoholic fatty liver disease tends to increase with age, and thus, aging and lipid metabolism in the liver may be closely related (Amarapurkar et al., 2007). In addition, evidence suggests that increased oxidative stress due to various factors leads to increased lipid accumulation in the liver, while decreased oxidative stress has a lipid‐lowering effect in hepatocytes (Hassan et al., 2014; Kharroubi et al., 2015; Silva et al., 2015).

Lipid supply to liver tissue consists of three main pathways: dietary intake, peripheral lipolysis, and de novo lipogenesis (Cogger, Hilmer, & Svistounov, 2011). Fatty liver occurs when the lipid supply exceeds the hepatic lipid removal. In many previous studies, triglyceride (TG) and cholesterol metabolism disorders and accumulation have been reported to be closely related to aging (Kim et al., 2015; Wang et al., 2013). For example, in the senescent‐associated mouse, the cholesterol content in the liver was increased compared with control mice (Kuhla, Blei, Jaster, & Vollmar, 2011). In this study, we investigated the mechanisms for the increase in cholesterol accumulation during aging. We found that the increased ROS in aging plays an important role for the accumulation of cholesterol in the liver by increasing cholesterol uptake and cholesterol synthesis via increasing glucose uptake.

## 2 RESULTS

### 2.1 Serum lipid profiles during aging

**Figure 1:**
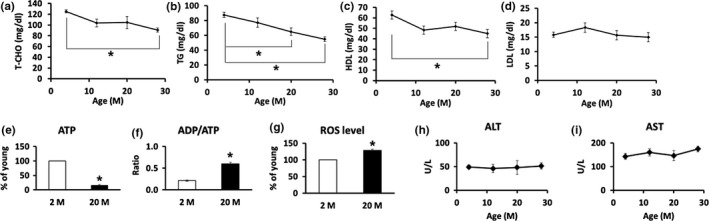
Changes in lipid concentration and liver function in mice at different ages (*n* = 8–10/group). (a) Total cholesterol (T‐CHO). (b) TG. (c) HDL cholesterol. (d) LDL cholesterol in serum. *p < 0.05. (e, f) Primary hepatocytes were isolated from young (2‐month‐old) and old (20‐month‐old) mice, and (e) ATP levels, (f) ADP/ATP ratios, and (g) ROS levels were measured. (h, i) Serum, (h) ALT levels, and (i) AST levels were measured. *p < 0.05 vs. 2 M

To determine changes in serum lipid concentration with aging, total cholesterol, TG, and high‐density lipoprotein (HDL) cholesterol and low‐density lipoprotein (LDL) cholesterol levels in serum of different ages of C57BL/6 mice (4, 12, 20, 28 months old) were monitored. Total cholesterol (Figure 1a, *p* = 0.000002) and TG (Figure 1b, *p* = 0.000001 4 months vs. 28 months) decreased significantly with increasing age. HDL cholesterol, known as “good” cholesterol, significantly decreased with age (Figure 1c, *p* = 0.00001), but LDL cholesterol, known as “bad” cholesterol, was not affected by aging (Figure 1d).

### 2.2 Reduced ATP production and increased ROS production in hepatocytes of aged mice

In order to investigate changes in hepatic function with aging, primary hepatocytes were isolated from young (2‐month‐old) and aged (20‐month‐old) mice, and the ATP levels were measured as an indicator of mitochondrial function. ATP levels in hepatocytes of 20‐month‐old mice were significantly reduced compared with that of 2‐month‐old mice (Figure 1e, *p* = 0.01). In addition, the ADP/ATP ratio was significantly increased in 20‐month‐old mice compared with 2‐month‐old mice (Figure 1f, *p* = 0.044). The level of ROS, which play a major role in functional deterioration during aging, in hepatocytes of 20‐month‐old mice, was significantly increased as compared with 2‐month‐old mice (Figure 1g, *p* = 0.009). However, the serum levels of serum aspartate transaminase (ALT; Figure 1h) and alanine transaminase (AST; Figure 1i), indexes of hepatotoxicity, did not show significant changes with age. These results indicate that liver mitochondrial function declined with age without significant liver damage.

### 2.3 Increased cholesterol accumulation in the liver of aged mice

**Figure 2:**
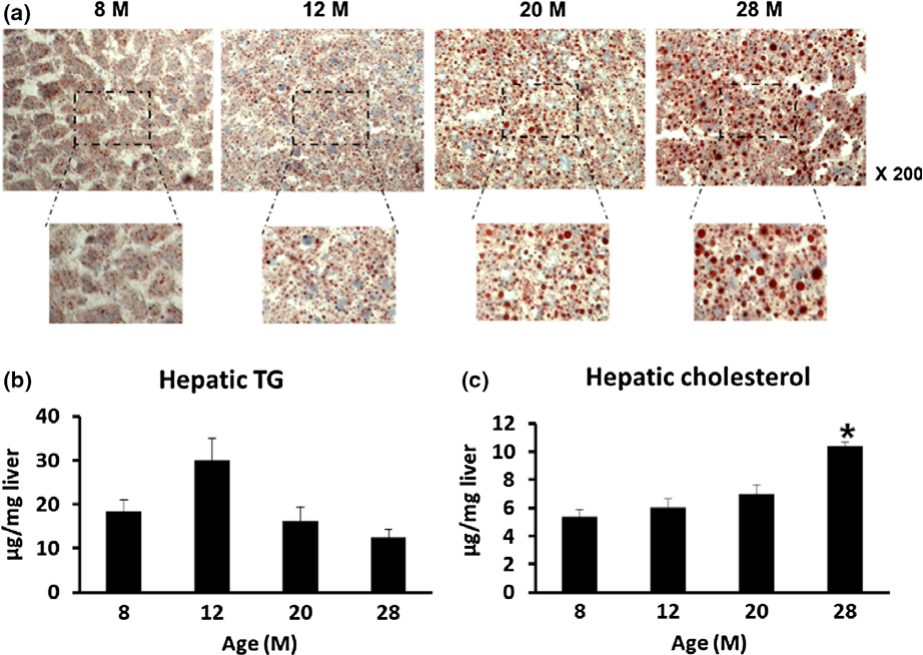
Changes in lipid accumulation in liver tissues at different ages of mice (*n* = 8–10/group). (a) Representative image of Oil Red O staining of liver tissue (Magnification; ×200). (b, c) The Folch method was used to separate lipids from liver tissue of various ages and to measure (b) hepatic TG levels and (c) hepatic cholesterol levels. *p < 0.05 vs. 8 M

In order to investigate the changes in fat accumulation in liver tissues during aging, liver sections from different ages of mice were stained with Oil Red O. As the age increased, the Oil Red O‐stained area was increased, indicating that accumulation of fat in the liver increased with aging (Figure 2a). When we analyzed TG levels in liver tissue, we found that there were no significant differences among ages (Figure 2b). However, hepatic cholesterol levels increased with age, with cholesterol levels in 28‐month‐old mice being significantly increased compared to 8‐month‐old mice (Figure 2c, *p* = 0.0003).

### 2.4 Increased cholesterol uptake and glucose uptake in hepatocytes of aged mice

**Figure 3:**
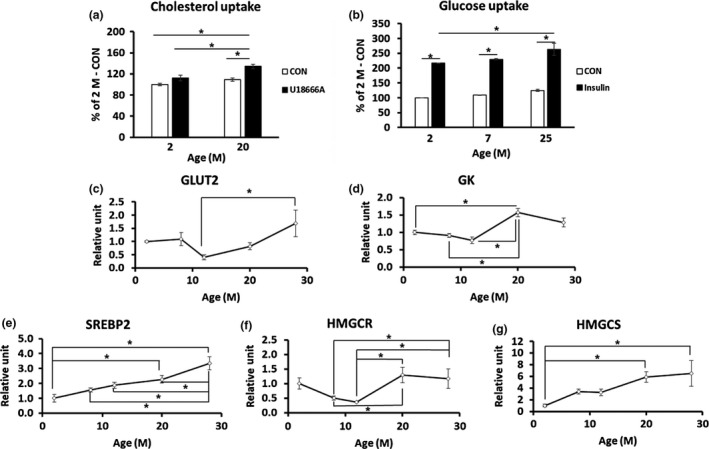
Cholesterol uptake and glucose uptake cholesterol synthesis‐related gene expression were increased in aged mice (*n* = 3/group). (a, b) Primary hepatocytes were isolated from mouse livers at various ages, and cholesterol and glucose uptake were measured. (a) Cholesterol uptake was measured in the absence (CON) or presence of 1.25 μM U18666A, a cholesterol uptake inducer. (b) Glucose uptake was measured in the absence (CON) or presence of 1 μM insulin*.* (c, d) Total RNA was isolated from mouse livers at various ages, and qRT–PCR was carried out (*n* = 8–10/group). (c) GLUT2; (d) GK; (e) SREBP2; (f) 3‐hydroxy‐3‐methylglutaryl‐CoA reductase (HMGCR); (g) 3‐hydroxy‐3‐methylglutaryl‐CoA synthase 1 (HMGCS). *p < 0.05

As we found that hepatic cholesterol was significantly increased at 28 months, we first checked whether cholesterol uptake in hepatocytes was changed in aged mice. Cholesterol uptake was significantly increased in primary hepatocytes of 20‐month‐old mice compared with 2‐month‐old mice. When the hepatocytes were treated with U18666A, a cholesterol uptake stimulator, cholesterol uptake was further increased in 20‐month‐old mice (Figure 2a). Insulin‐stimulated glucose uptake in primary hepatocytes from 25‐month‐old mice was significantly increased over that of 2‐month‐old mice (Figure 3b). mRNA expression of glucose transporter 2 (GLUT2; Figure 3c, *p* = 0.047), which plays an important role in glucose uptake, was observed to increase at 28 months compared with 12 months, and glucokinase (GK; Figure 3d, *p* = 0.009), a gene related to glycolysis, was also observed to increase at 20 months compared with 2 months. In addition, the mRNA expression levels of genes involved in the synthesis of cholesterol in hepatocytes such as sterol regulatory element‐binding protein 2 (SREBP2; Figure 3e, *p* = 0.006) and 3‐hydroxy‐3‐methylglutaryl‐CoA synthase 1 (HMGCS; Figure 3g, *p* = 0.002) were increased in 20‐month‐old mice, compared to the case for 2‐month‐old mice, and that of 3‐hydroxy‐3‐methylglutaryl‐CoA reductase (HMGCR) was increased in 20‐month‐old mice, compared to the case for 8‐month‐old mice (Figure 3f, *p* = 0.016). These results suggest that increased uptake of glucose in aged mice might contribute to a source for cholesterol synthesis through the glycolysis process.

### 2.5 Increased cholesterol and triglyceride synthesis by induction of ROS in HepG2 cells and primary hepatocytes

**Figure 4:**
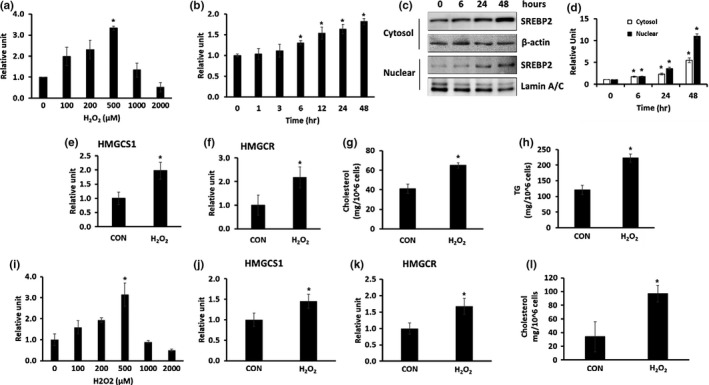
(a–h) Induction of ROS by H_2_O_2_ treatment increased cholesterol and triglyceride synthesis in HepG2 cells (*n* = 3/group). (a) HepG2 cells were untreated (CON) or treated with various concentrations of H_2_O_2_ for 24 hr, and the expression of SREBP2 mRNA was determined by qRT–PCR. (b–d) HepG2 cells were untreated (CON) or treated with 500 µM H_2_O_2_ for various times. (b) The expression of SREBP2 mRNA was determined by qRT–PCR. (c) SREBP2 protein expression in the cytosol and nucleus was determined using western blotting. (d) Quantitative analysis of (c). (e, f) HepG2 cells were treated with 500 µM H_2_O_2_ for 48 hr and harvested. (e) Identification of HMGCS mRNA expression using qRT–PCR. (f) Identification of HMGCR mRNA expression using qRT–PCR (g) Intracellular TG level. (h) Intracellular cholesterol level *p < 0.05 vs. CON. (i–l) Induction of ROS by H_2_O_2_ treatment increased cholesterol synthesis in primary hepatocytes (*n* = 3/group). (i) Primary hepatocytes were left untreated (CON) or treated with various concentrations of H_2_O_2_ for 48 hr, and the expression of SREBP2 mRNA was determined by qRT–PCR. (j–l) Primary hepatocytes were treated with 500 μM H_2_O_2 _for 48 hr and harvested. (j) HMGCS mRNA expression and (k) HMGCR mRNA expression determined by qRT–PCR. (l) Intracellular cholesterol level *p < 0.05 vs. CON

To investigate the detailed mechanism of cholesterol accumulation by aging, HepG2 cells, a human hepatocellular carcinoma cell (HCC) line, were treated with various concentrations of H_2_O_2_ for 24 hr, and mRNA expression of SREBP2, a key regulator of cholesterol synthesis, was measured. Treatment with 500 μM H_2_O_2_ showed the highest expression of SREBP2 without cytotoxicity (Figure 4a, *p* = 0.049). Treatment of HepG2 cell with 500 μM H_2_O_2_ for different incubation times (0–48 hr) showed that SREBP2 mRNA (Figure 4b) and protein (Figure 4c,d) expression were increased in a time‐dependent manner. In addition, mRNA expression of HMGCS (Figure 4e, *p* = 0.021) and HMGCR (Figure 4f, *p* = 0.048), which are involved in cholesterol synthesis, was increased. When we measured TG and total cholesterol content in HepG2 cells after treatment 500 μM H_2_O_2_ for 48 hr, we found that both cholesterol (Figure 4g, *p* = 0.043) and TG (Figure 4h, *p* = 0.032) were significantly increased by H_2_O_2_ treatment (Figure 3g,h). These results suggest that H_2_O_2_ treatment of HepG2 cells can be used as a model for ROS‐induced lipid accumulation as observed in aging.

To validate the obtained results using HepG2 cells, we isolated primary hepatocytes from 2‐month‐old mice, treated them with various concentrations of H_2_O_2_ for 48 hr, and measured the mRNA expression of SREBP2. Treatment with 500 μM H_2_O_2_ showed the highest expression of SREBP2 without cytotoxicity (Figure 4i, *p* = 0.039). Thus, we chose 500 μM H_2_O_2 _for further experiments. Treatment of primary hepatocytes with 500 μM H_2_O_2_ for 48 hr increased the mRNA expression of HMGCS (Figure 4j, *p* = 0.001) and HMGCR (Figure 4k, *p* = 0.045). When we measured the total cholesterol content in primary hepatocytes, we found that intracellular cholesterol levels were significantly increased by H_2_O_2_ treatment (Figure 4l, *p* = 0.039), similar to the results found in case of HepG2 cells.

### 2.6 Increased expression of caveolin‐1 and LDLR in H_2_O_2_‐treated HepG2 cells and the increased cholesterol uptake in H_2_O_2_‐treated primary hepatocytes

**Figure 5:**
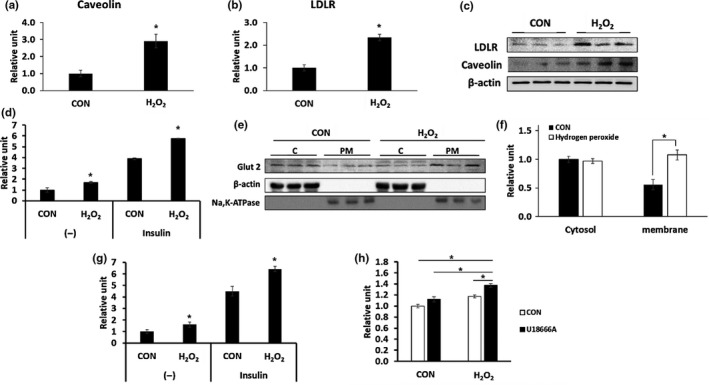
(a–c) mRNA and protein expression of caveolin‐1 and LDLR was increased in H_2_O_2_‐treated HepG2 cells. HepG2 cells were untreated (CON) or treated with 500 µM H_2_O_2 _for 48 hr and harvested (*n* = 3/group). (a) Identification of caveolin‐1 mRNA expression using qRT–PCR. (b) Identification of LDLR mRNA expression using qRT–PCR. (c) Identification of caveolin‐1 and LDLR protein expression using western blotting. (d–f) Increase in glucose uptake in H_2_O_2_‐treated HepG2 cells. HepG2 cells were untreated (CON) or treated with 500 µM H_2_O_2_ for 48 hr and harvested (*n* = 3/group). (d) HepG2 cells were treated without (−) or with of 1 μM insulin, and a glucose uptake assay was performed. (e) Identification of GLUT2 in the cytosol and plasma membrane using western blotting. (f) Quantitative analysis of (e). *p < 0.05 vs. CON. (g, h) Primary hepatocytes were treated with 500 µM H_2_O_2 _for 48 hr, and glucose uptake and cholesterol uptake were measured (*n* = 3/group). (g) Glucose uptake was measured in the absence or presence of 1 μM insulin*.* (h) Cholesterol uptake was measured in the absence or presence of 1.25 μM U18666A, an inducer of cholesterol uptake. *p < 0.05

To investigate whether cholesterol uptake can be increased by ROS induction, HepG2 cells were treated with 500 μM H_2_O_2_ for 48 hr. We then examined mRNA and protein levels of caveolin‐1 and low‐density lipoprotein receptor (LDLR), which are involved in cholesterol uptake. The expression of caveolin‐1 (*p* = 0.032) and LDLR (*p* = 0.01) mRNA and protein levels increased in H_2_O_2_‐treated cells (Figure 5). These results suggest that increased expression of caveolin‐1 and LDLR can contribute to the increased cholesterol uptake in H_2_O_2_‐treated HepG2 cells. We then examined cholesterol uptake in primary hepatocytes. Treatment of primary hepatocytes from 2‐month‐old mice with H_2_O_2_ significantly increased cholesterol uptake compared with the non‐treated cells. When the hepatocytes were treated with U18666A, a cholesterol uptake stimulator, cholesterol uptake was further increased in H_2_O_2_‐treated primary hepatocytes (Figure 5h).

### 2.7 Increased glucose uptake and related factors in H_2_O_2_‐treated HepG2 cells and primary hepatocytes

Glucose taken up in liver cells is the source for cholesterol synthesis (Donnelly et al., 2005). To investigate whether glucose uptake is increased by ROS induction, a glucose uptake assay was performed after treatment with 500 μM H_2_O_2_ in HepG2 cells. H_2_O_2_ treatment increased both basal and insulin‐stimulated glucose uptake (Figure 5d). As glucose uptake was increased, we checked the translocation of Gut2 protein. We found that GLUT2 expression level in the cytosol was not changed during H_2_O_2_ treatment, but GLUT2 expression level in the plasma membrane was significantly increased (Figure 5e,f). These results suggest that glucose uptake may be increased due to the increased translocation of GLUT2 to the membrane under conditions of ROS induction. Similarly, both basal and insulin‐stimulated glucose uptake were significantly increased by H_2_O_2_ treatment in primary hepatocytes (Figure 5g).

### 2.8 Glucose as a source of cholesterol synthesis in H_2_O_2_‐treated HepG2 cells and primary hepatocytes

**Figure 6:**
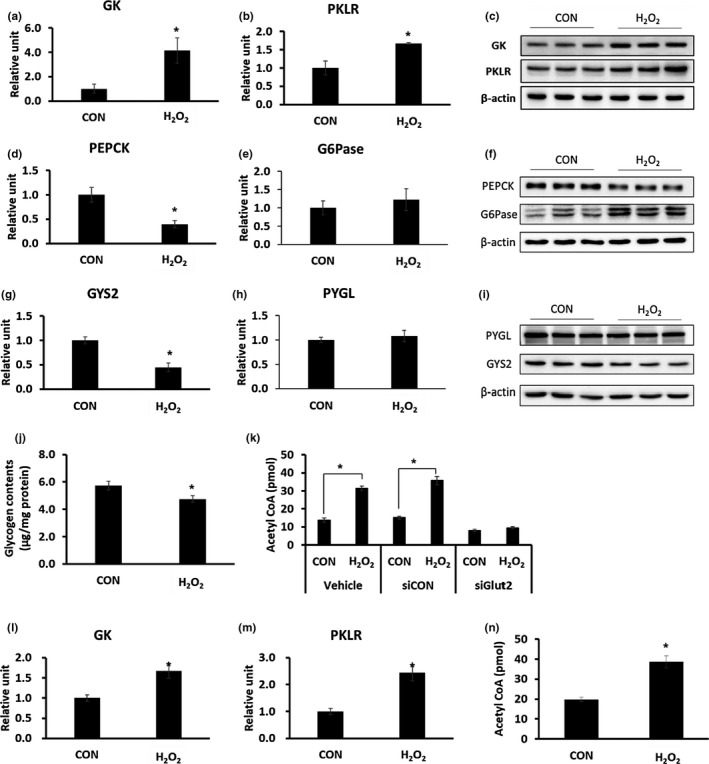
Changes in glucose metabolism‐related factors by ROS induction. (a–k) HepG2 cells were untreated (CON) or treated with 500 µM H_2_O_2_ for 48 hr and harvested. (*n* = 3/group). (a, b, d, e, g, h) Measurement of gene expression by qRT–PCR. (c, f, i) Measurement of protein levels using western blotting. (j) Measurement of intracellular glycogen levels. (k) After GLUT2 knockdown using GLUT2 siRNA, HepG2 cells were untreated (CON) or treated with 500 µM H_2_O_2_ for 48 hr and harvested; intracellular acetyl‐CoA levels were measured. *p < 0.05 vs. CON. (l–n) Primary hepatocytes were treated with 500 µM H_2_O_2 _for 48 hr and harvested (*n* = 3/group). (l) Measurement of mRNA expression of GK by qRT–PCR. (m) Measurement of mRNA expression of PKLR by qRT–PCR. (n) Intracellular acetyl‐CoA levels were measured. *p < 0.05 vs. CON

Although glucose uptake was increased by H_2_O_2_ treatment, it was not known whether glucose uptake is used as a source for cholesterol synthesis in HepG2 cells. We first checked the expression of molecules involved in glycolysis. The mRNA and protein levels of GK (*p* = 0.049) and pyruvate kinase isozymes R/L (PKLR; *p* = 0.038), which are related to glycolysis, were significantly increased by H_2_O_2_ treatment (Figure 6a–c). We then examined the expression of genes involved in the early stages of gluconeogenesis. mRNA and protein levels of phosphoenolpyruvate carboxykinase (PEPCK; *p* = 0.008) were significantly reduced by H_2_O_2_ treatment (Figure 6d,f). In the case of glucose 6‐phosphatase (G6pase), which is involved in the last stage of gluconeogenesis, protein levels and mRNA expression were not significantly different (Figure 6e,f). The mRNA and protein level of glycogen synthase 2 (GYS2; *p* = 0.037), a factor related to glycogen synthesis, were significantly decreased by H_2_O_2_ treatment, whereas PYGL, a factor associated with glycogen degradation, was not changed (Figure 6g–i). The hepatic glycogen content was measured to determine whether the expression of the genes related to glycogen synthesis and degradation coincided with the levels of hepatic glycogen. The intracellular glycogen concentration decreased in H_2_O_2_‐treated HepG2 cells (Figure 6j, *p* = 0.004). The level of acetyl‐CoA, the end product of glycolysis, was significantly increased during H_2_O_2_ treatment. However, when glucose uptake was inhibited by knockdown of GLUT2 gene using siRNA, there was no increase in the accumulation of acetyl‐CoA by H_2_O_2_ treatment (Figure 6k). We also confirmed that the mRNA expression levels of GK (*p* = 0.044) and PKLR (*p* = 0.035) increased significantly (Figure 6l,m) in primary hepatocytes after H_2_O_2_ treatment, as found in case of the HepG2 cells. Acetyl‐CoA levels were also significantly increased in primary hepatocytes after H_2_O_2_ treatment (Figure 6n, *p* = 0.004). These results show that increased uptake of glucose by ROS could be used as a source of cholesterol synthesis through an increased glycolysis process.

## 3 DISCUSSION

Aging is highly related to the disorders of glucose and lipid metabolism. (Mc Auley & Mooney, 2015; Salmon, 2012), and hepatic lipid accumulation can contribute to aging‐related diseases, including fatty liver, liver cirrhosis, and metabolic syndrome (Sheedfar, Biase, Koonen, & Vinciguerra, 2013). Hepatic lipid deposition is strictly controlled by a variety of factors including dietary lipid intake, circulating lipid levels, glucose uptake, lipid synthesis, hepatic lipid oxidation, and liver lipid release. (Gong, Tas, Yakar, & Muzumdar, 2017). Therefore, changes in liver lipid content might be due to the changes in glucose and lipid metabolism. The mechanisms for TG accumulation in the liver in aging have been extensively studied. (Guan et al., 2013; Xiong et al., 2014). However, there has been little research on the hepatic accumulation of cholesterol following aging.

In this study, a decrease in total serum cholesterol levels was observed in aged mice due to the decrease in HDL cholesterol. Serum TG levels were also decreased with aging. These results are consistent with the previous report that levels of free fatty acid in serum of aged mice are increased, but TG levels were reduced (Houtkooper et al., 2011).

ATP levels, which indirectly recognize the metabolic function of the liver, were greatly reduced, and ROS levels were significantly increased in hepatocytes of aged mice. These results indicated that hepatic mitochondrial function is damaged by aging. The mitochondria are intracellular organelles that play an important role in glucose and lipid metabolism, and thus, dysfunction of mitochondria in hepatocytes of aged mice might contribute to liver metabolic abnormalities. Our aging animal model showed accumulation of fat in liver evidenced by the increased Oil Red O staining. Our results showed that hepatic cholesterol, but not hepatic TG, increased with age. This phenomenon has also been reported in senescence‐accelerated prone mice (SAMP8), an aging animal model (Kuhla et al., 2011). Therefore, we aimed to investigate the mechanisms of hepatic cholesterol accumulation by aging.

First, we found that cholesterol uptake was increased in primary hepatocytes isolated from aged mice. Another pathway for cholesterol accumulation is de novo synthesis from glucose. The uptaken glucose is oxidized to pyruvate through the glycolysis process and is converted to citrate through the Krebs cycle of mitochondria. The citrate is cleaved to form acetyl‐CoA for elongation of fatty acid chains. Once the chain reaches 16 carbon lengths, palmitate is released and then oxidized or stored as TG. The acetyl‐CoA can also be used to synthesize cholesterol through the action of HMGCS and HMGCR (Vock, Doring, & Nitz, 2008). The expression of SREBP2, HMGCR, and HMGCS, which are related to cholesterol synthesis, was increased with aging. Glucose uptake, which can contribute to the production of acetyl‐CoA, was increased, and in parallel, cholesterol synthesis was also found to be increased in primary hepatocytes of aged mice. These results suggest that cholesterol content increases in the liver of aged mice through direct uptake of cholesterol and de novo synthesis.

To investigate the mechanism of cholesterol metabolism disorder in aging, in vitro analysis was performed using HepG2 cells, a human hepatocyte HCC cell line. The ROS levels were significantly increased in primary hepatocytes isolated from senescent mice, in agreement with other findings (Davalli, Mitic, Caporali, Lauriola, & D'Arca, 2016). To mimic ROS‐induced aging in cells, we treated HepG2 cells with H_2_O_2_. Treatment of HepG2 cells with H_2_O_2_ increased the expression of SREBP2, the master regulator of cholesterol synthesis, and increased its translocation into the nucleus. The expression of HMGCR and HMGCS was also increased. In addition, H_2_O_2_ treatment increased the levels of TG and cholesterol in HepG2 cells. Although there was no increase in hepatic TG in the aging animal model, increase in TG accumulation was observed in the H_2_O_2_‐treated HepG2 cells. These differences may be due to the metabolic regulation mechanism of the whole body.

The expression of mRNA and protein for caveolin‐1 and LDLR, which are involved in cholesterol uptake (Ikonen, 2006), was significantly increased in H_2_O_2_‐treated HepG2 cells. An increase in cholesterol uptake was observed in hepatocytes for aged mice. These results suggest that increased ROS can induce the expression of these genes and contribute to the increase in cholesterol uptake.

Glucose uptake and the translocation of GLUT2, a factor associated with glucose uptake, to the cell membrane, were increased in HepG2 cells treated with H_2_O_2_. This result indicates that glucose uptake can also be increased by ROS. De novo synthesis of hepatic lipid is regulated independently by insulin and glucose (Koo, Dutcher, & Towle, 2001). In the postprandial state, glucose is taken up into hepatocytes through GLUT2 and phosphorylated to glucose‐6‐phosphate by hepatic GK (Massa, Gagliardino, & Francini, 2011), which undergoes glycolysis to produce acetyl‐CoA, a source for lipid synthesis (Browning & Horton, 2004). In this study, the hepatic GK and PKLR, genes related to glycolysis, were significantly increased and acetyl‐CoA, levels were also increased in H_2_O_2_‐treated HepG2 cells. Therefore, ROS increases glucose uptake and promotes the glycolysis process and thereby increasing fat synthesis in the aged liver.

Glucose‐6‐phosphate can also be used for glycogen synthesis and storage depending on the systemic metabolic status (von Wilamowitz‐Moellendorff et al., 2013). Therefore, we also examined expression of glycogen‐related genes and glycogen content in H_2_O_2_‐treated HepG2 cells. The expression of GYS2, which is involved in glycogen synthesis in the liver, was decreased, and the intracellular glycogen level was also decreased by H_2_O_2_ treatment. The expression of PYGL, which is involved in glycogen degradation, was not changed. Therefore, it is possible that increased glucose‐6‐phosphate originating from glucose uptake might be used as a source of lipid synthesis rather than glycogen synthesis. The expression of gluconeogenesis‐related factors was also confirmed, but the expression of PEPCK, which acts as a rate‐limiting enzyme in the early stage (Chia, Liong, Ton, & Kadir, 2012), was significantly reduced. Finally, when GLUT2 was knocked down, there was no increase in acetyl‐CoA level due to H_2_O_2_ treatment, indicating that increased fat synthesis occurred through increased glucose uptake by ROS.

HepG2 cell is a cancer cell line and may thus show the Warburg effect (Vander Heiden, Cantley, & Thompson, 2009); this may cause metabolic changes. Therefore, we used primary hepatocytes to confirm the results found in HepG2 cells. Similar to the phenomenon seen in primary hepatocytes isolated from old mice or in HepG2 cells treated with H_2_O_2_, the primary hepatocytes (from young mice) treated with H_2_O_2_ showed an increased cholesterol uptake and glucose uptake. In addition, the mRNA expression of genes related with glycolysis and cholesterol synthesis, and intracellular cholesterol accumulation was also increased, as observed in case of the HepG2 cells. These results suggest that the metabolic mechanisms found in HepG2 cells are similar to those in primary hepatocytes.

There are many reports that ROS play an important role in various metabolic diseases associated with aging (Bonomini et al., 2015; Roberts & Sindhu, 2009; Salminen, Ojala, Kaarniranta, & Kauppinen, 2012). We found that ROS contribute to increased cholesterol and glucose uptake into the cells and the increase in cholesterol synthesis from glucose, resulting in an increase in cholesterol accumulation in the liver with aging.

## 4 EXPERIMENTAL PROCEDURES

### 4.1 Animals

Male C57BL/6 mice were supplied by the Korea Research Institute of Bioscience and Biotechnology (Daejeon, Korea). Animals were maintained in the specific‐pathogen‐free (SPF) animal facilities at the Lee Gil Ya Cancer and Diabetes Institute, Gachon University, under a 12‐hr light, 12‐hr dark photoperiod. All animal experiments were carried out under a protocol approved by the Institutional Animal Care and Use Committee at Lee Gil Ya Cancer and Diabetes Institute, Gachon University. All animals were fed ad libitum, with free access to water and a normal chow diet (PicoLab Rodent Diet 20 5053; 20% protein from plant and animals sources, 4.5% (wt/wt) fat, 0.02% (wt/wt) cholesterol, no casein, no sodium cholate; LabDiet, MO, USA).

### 4.2 Primary hepatocyte isolation

C57BL/6 male mice were anesthetized with ketamine hydrochloride (40 mg/kg) by intraperitoneal injection, and their livers were perfused with a solution of 142 mM NaCl, 6.7 mM KCl, 10 mM HEPES, and 2.5 mM EGTA (pH 7.4). This solution was replaced by 0.5 mg/ml collagenase and 10 mg/ml albumin in 66.7 mM NaCl, 6.7 mM KCl, 10 mM HEPES, 4.8 mM CaCl, pH 7.6. The perfused livers were harvested, rinsed, and disaggregated. After centrifugation, cells were suspended in an appropriate volume of the culture medium (HepatoZYME‐SFM, Gibco BRL, MA, USA).

### 4.3 Cell culture

HepG2 (ATCC, Rockville, MD) cells were maintained at subconfluence at 37°C with 5% CO_2_. The cells were grown in DMEM with 10% FBS containing 100 units/ml of penicillin and streptomycin. To establish the aging cell model, HepG2 cells and primary hepatocytes were treated with 500 μM H_2_O_2_ for 48 hr.

### 4.4 Quantification of lipid level of serum, liver tissue, and cells

Blood samples were centrifuged at 3,000 *g* for 20 min, and serum was collected. Serum levels of total cholesterol, TG, LDL cholesterol, and HDL cholesterol were measured using Beckman Coulter AU680 chemistry analyzer (Beckman Coulter, Inc., CA, USA). Total lipids were separated from the liver tissue or cells using the Folch method (Folch, Ascoli, Lees, Meath, & Le, 1951). TG and total cholesterol content was measured using a commercial enzymatic kit (Asan Pharmaceutical Company, Seoul, Korea).

### 4.5 ATP level and ADT/ATP ratio measurements

The ATP levels and the ADP/ATP ratio of primary hepatocytes were measured using the ADP/ATP Ratio Assay Kit (Abcam, MA, USA).

### 4.6 Measurement of serum ALT/AST level

Serum ALT/AST was measured using a commercial enzymatic kit (Asan Pharmaceutical Company, Seoul, Korea).

### 4.7 Measurement of glycogen and acetyl‐CoA level of HepG2 cells

Glycogen and acetyl‐CoA levels were determined using a commercial enzymatic kit (Biovision).

### 4.8 ROS detection

For quantification of intracellular ROS levels, primary hepatocytes were loaded with 10 μM 2′, 7′‐dichlorodihydrofluorescein diacetate (Molecular Probes, OR, USA) for 30 min at 37°C, 5% CO_2_ in phosphate‐buffered saline. Cells were collected, washed twice with PBS, and suspended in 500 μl PBS. Fluorescent intensity was measured using FACSCalibur (BD Biosciences, CA, USA) and analyzed by CellQuest Pro 5.2 according to the manufacturer's protocol.

### 4.9 Oil Red O staining

Liver pieces were embedded in optimal cutting temperature compound. Frozen liver sections were cut at 10 μm thickness, fixed with 10% buffered formalin, dehydrated with 100% propylene glycol, and stained with 0.5% Oil Red O for 30 min at 55°C. Sections were washed repeatedly with 85% propylene glycol followed by distilled water and stained with hematoxylin. Lipid droplets were stained red.

### 4.10 Glucose and cholesterol uptake assays

For glucose uptake assay, the HepG2 cells or primary hepatocytes were added to the wells of a 96‐well plate (1 × 10^4^ cells/well) and incubated for 24 hr in growth medium. The growth media were then replaced with medium not containing FBS, and the plates were incubated for another 24 hr. The medium was replaced with Krebs–Ringer–phosphate–HEPES buffer containing 2% bovine serum albumin. Glucose uptake was determined using a Glucose Uptake Colorimetric Assay Kit (Biovision, CA, USA), following the manufacturer's instructions. For cholesterol uptake assay, the HepG2 cells or primary hepatocytes were added to the wells of a 96‐well plate (1 × 10^4^ cells/well) and incubated for 24 hr in growth medium. Cells were treated with serum‐free culture medium containing 20 μg/ml NBD cholesterol and incubated for 24 hr. 1.25 μM U18666A was used as a positive control. Cholesterol uptake was determined using a Cholesterol Uptake Cell‐Based Assay Kit (Cayman Chemical, MI, USA), following the manufacturer's instructions.

### 4.11 Western blotting

Cells were harvested, and total protein was extracted or membrane/cytosol fractionation was carried as described previously (Yamamoto, Yamashita, Yoshioka, Nishiumi, & Ashida, 2016). Proteins (30–50 μg) were resolved by sodium dodecyl sulfate–polyacrylamide gel electrophoresis, transferred onto membranes, and blocked with 5% nonfat dry milk in Tris‐buffered saline containing Tween 20. Membranes were incubated with specific primary antibodies and visualized by incubating with horseradish peroxidase (HRP)‐conjugated secondary antibodies. Chemiluminescence was detected by LAS‐4000 (Fuji Film, Tokyo, Japan) after adding Immobilon Western Chemiluminescent HRP Substrate (Millipore, St. Charles, MO). Antibodies against SREBP2, LDLR, GLUT2, GK, PEPCK, G6pase, PKLR, and GYS2 were obtained from Santa Cruz Biotechnology Inc. (CA, USA). Antibodies against β‐actin, Lamin A/C, and Na/K‐ATPase were obtained from Cell Signaling Technology (MA, USA). Antibodies against caveolin were obtained from BD Biosciences (CA, USA). Antibodies against PYGL and HRP‐conjugated secondary antibodies were obtained from Bethyl Laboratories (TX, USA).

### 4.12 Quantitative real‐time RT–PCR analysis

Total RNA was extracted from the liver tissue or cells using TRIzol reagent (Invitrogen Corp., CA, USA), following the manufacturer's instructions, and cDNA was synthesized using a PrimeScript 1st strand cDNA synthesis kit (Takara Bio Inc., Kyoto, Japan). Quantitative real‐time RT–PCR (qRT–PCR) was performed using the SYBR Premix Ex Taq II, ROX plus (Takara Bio Inc.), and the Prism 7900HT sequence detection system (Applied Biosystems, Foster City, CA, USA). PCR was carried out for 40 cycles (2 min at 50°C, 10 min at 95°C°, and 40 cycles of 10 s at 95°C and 1 min at 60°C). The primer sequences used are shown in Table 1. The relative copy number was calculated using the threshold crossing point (*C_t_*) as calculated by ΔΔ*C_t_*.

**Table 1:** Primers used for quantitative real‐time PCR

Gene	Forward/Reverse primers
*mCyclophilin*	5′‐TGGAGAGCACCAAGACAGACA 5′‐TGCCGGAGTCGACAATGAT
*mSREBP2*	5′‐GCGTTCTGGAGACCATGGA 5′‐ACAAAGTTGCTCTGAAAACAAATCA
*mGK*	5′‐CCCTGAGTGGCTTACAGTTC 5′‐ACGGATGTGGAGTGTTGAAGC
*mGLUT2*	5′‐CCTCAAGAGGTAATAATATCCC 5′‐CCATCAAGAGGGCTCCAGTC
*mHMGCR*	5′‐CTTGTGGAATGCCTTGTGATTG 5′‐AGCCGAAGCAGCACATGAT
*mHMGCS*	5′‐TGCACGGATCGTGAAGACA 5′‐TGCACGGATCGTGAAGACA
*hCyclophilin*	5′‐TGCCATCGCCAAGGAGTAG 5′‐TGCACAGACGGTCACTCAAA
*hSREBP2*	5′‐CCGCCTGTTCCGATGTACAC 5′‐TGCACATTCAGCCAGGTTCA
*hCaveolin‐1*	5′‐TTCCGGGCTTCATCTGGCAAC 5′‐GCTCAGCCCTATTGGTCCACTTTA
*hLDLR*	5′‐CAGTCTGGAGGATGACGTGG 5′‐ACTGTCCGAAGCCTGTTCTG
*hGK*	5′‐CCGCAGCGAGGACGTAAT 5′‐CTTGTACACGGAGCCATCCA
*hPKLR*	5′‐AGGCGTGAAGAGGTTTGATGA 5′‐CCCCCGTGCCACCAT
*hPEPCK*	5′‐TGAAAGGCCTGGGGCACAT 5′‐TTGCTTCAAGGCAAGGATCTCT
*hG6pase*	5′‐TCATCTTGGTGTCCGTGATCG 5′‐TTTATCAGGGGCACGGAAGTG
*hGYS2*	5′‐CTGACAGGGCAGATACCGTC 5′‐ACCAGGCTTTGGTAGCTTCTC
*hPYGL*	5′‐CCACCCGCGACTACTACTTC 5′‐CAAGCTTGGGGCACTTGTC

### 4.13 siRNA knockdown of GLUT2

HepG2 cells (2 × 10^5^ cells) were transfected with GLUT2‐siRNA (sc‐35495; 10 pmol/L) or control siRNA (sc‐44230, 10 pmol/L) (Santa Cruz Biotechnology Inc.) using RNAiMAX (Invitrogen) following the manufacturer's instructions.

### 4.14 Statistical analyses

All data are expressed as mean ± standard error of at least three independent experiments. Data were analyzed using analysis of variance followed by post hoc analysis using the Tukey range test (GraphPad Prism 7.04 statistical software). *p*‐Values <0.05 were considered statistically significant.

## CONFLICT OF INTERESTS

The authors have declared that no competing interests exist.

## AUTHOR’S CONTRIBUTION

The authors’ responsibilities were as follows—H.‐S.J. conceived and designed the study, and wrote the manuscript. E.S. contributed to the design of the study, performed the experiments, and wrote the manuscript. H.K., H.C., and W.C. performed the experiments.
